# Rotavirus A-specific single-domain antibodies produced in baculovirus-infected insect larvae are protective *in vivo*

**DOI:** 10.1186/1472-6750-12-59

**Published:** 2012-09-07

**Authors:** Silvia Gómez-Sebastián, Maria C Nuñez, Lorena Garaicoechea, Carmen Alvarado, Marina Mozgovoj, Rodrigo Lasa, Alan Kahl, Andres Wigdorovitz, Viviana Parreño, José M Escribano

**Affiliations:** 1 Alternative Gene Expression S.L. (ALGENEX), Centro empresarial, Parque Científico y Tecnológico de la Universidad Politécnica de Madrid, Campus de Montegancedo, 28223, Pozuelo de Alarcón, Madrid, Spain; 2 Instituto de Virología, CICV y A–INTA, Buenos Aires, Argentina; 3 Departamento de Biotecnología, Instituto Nacional de Investigación y Tecnología Agraria y Alimentaria (INIA), Autovía A6, Km 7.5, 28040, Madrid, Spain

**Keywords:** Single-domain antibodies, Therapeutic molecule, Neutralization, Rotavirus A, Insect, Baculovirus, IBES®technology

## Abstract

**Background:**

Single-domain antibodies (sdAbs), also known as nanobodies or VHHs, are characterized by high stability and solubility, thus maintaining the affinity and therapeutic value provided by conventional antibodies. Given these properties, VHHs offer a novel alternative to classical antibody approaches. To date, VHHs have been produced mainly in *E. coli*, yeast, plants and mammalian cells. To apply the single-domain antibodies as a preventive or therapeutic strategy to control rotavirus infections in developing countries (444,000 deaths in children under 5 years of age) has to be minimized their production costs.

**Results:**

Here we describe the highly efficient expression of functional VHHs by the Improved Baculovirus Expression System (*IBES*® technology), which uses a baculovirus expression vector in combination with *Trichoplusia ni* larvae as living biofactories. Two VHHs, named 3B2 and 2KD1, specific for the inner capsid protein VP6 of Group A rotavirus, were expressed in insect larvae. The IBES® technology achieved very high expression of 3B2 and 2KD1, reaching 2.62% and 3.63% of the total soluble protein obtained from larvae, respectively. These expression levels represent up to 257 mg/L of protein extract after insect processing (1 L extract represents about 125 g of insect biomass or about 375 insect larvae). Larva-derived antibodies were fully functional when tested *in vitro* and *in vivo*, neutralizing Group A rotaviruses and protecting offspring mice against rotavirus-induced diarrhea.

**Conclusions:**

Our results open up the possibility of using insects as living biofactories (*IBES®* technology) for the cost-efficient production of these and other fully functional VHHs to be used for diagnostic or therapeutic purposes, thereby eliminating concerns regarding the use of bacterial or mammalian cells. To the best of our knowledge, this is the first time that insects have been used as living biofactories to produce a VHH molecule.

## Background

Conventional antibodies are complex molecules that consist of heavy and light chains. They contain an N-linked oligosaccharide attached to the second heavy-chain constant domain (CH2), which is essential for antibody functionality. Isolated heavy and light chains can retain antigen-binding specificity, but very frequently their affinity and solubility are reduced. Antibody production in heterologous expression systems is often cumbersome [1]. In this regard, a major scientific breakthrough was the discovery of heavy-chain antibodies. These are produced by camelids (bactrian camels, dromedaries, llamas and alpacas) and are functional antibodies devoid of light chains [2]. Camelid heavy-chain antibodies also lack the CH1 domain, which interacts with the light chain in conventional antibodies. Their single N-terminal domain (VHH) binds antigen without requiring domain pairing and it is the smallest domain with antigen-binding properties.

The use of VHHs in biotechnology has increased considerably thanks to their ease of manipulation, high affinity and stability and the well-established methods to generate antigen-specific VHH antibody libraries. Furthermore, some of these molecules remain functional after incubation at high temperature. This characteristic may be attributed to their efficient refolding after chemical or thermal denaturalization, which may occur as a result of increased hydrophilicity in their amino acid sequence [3]. Because of their small size (about 15 kDa) and the capacity of the extended CDR3 loop to penetrate very small sites, VHHs can also recognize active enzymatic sites [4]. Thus VHHs not only pass the renal filter, thus resulting in rapid blood clearance (serum half-life of about 2 h), but also show rapid tissue penetration.

VHH antibody fragments are emerging as new versatile reagents for the diagnosis and therapy of infectious diseases, including Rotavirus-induced diarrhea [5-7]. Rotavirus (RV) is estimated to cause 111 million episodes of gastroenteritis per year, resulting in 444,000 deaths in children under 5 years of age, and children in developing countries account for 82% of the deaths caused by this disease [8]. Currently, there are two licensed vaccines against RV-induced diarrhea in infants; however, no specific therapy is yet available.

RVs are classified into seven groups (A to G) on the basis of the antigenic variation of the inner capsid protein VP6, Group A (RV A) being the leading cause of acute gastroenteritis in infants. Group A RVs are further classified into P and G genotypes/serotypes on the basis of the genetic and antigenic variation of the outer layer proteins VP4 and VP7, both inducing the neutralizing antibodies associated with protection. This neutralization is due to the inhibition of virus binding to integrins [9]. Despite the different G and P serotypes, VP6 is a strongly conserved protein among all Group A RVs (90% amino acid homology). This structural virus protein is highly immunogenic and constitutes the target antigen of most immunodiagnostic tests for Group A RV detection.

Conventional antibodies directed to this protein do not show neutralizing activity *in vitro*[10]. However, inhibition of RV replication by a non-neutralizing RV VP6-specific IgA has been described [9]. Recently, it has also been demonstrated that VP6-specific VHHs produced in *E. coli* have broad RV neutralizing activity *in vitro* and their functionality was further confirmed by *in vivo* protection experiments in a neonatal mouse model [5]. The mechanism responsible for the broad neutralization of these single-domain antibodies *in vitro,* together with their protective properties in neonatal mice, is unknown. However, the effects of these antibodies could be related to their small size, which would allow a larger range of interactions with the inner capsid protein VP6 than conventional antibodies.

To date, VHHs have been produced in *E. coli*, yeast, plants and mammalian cells [1], and there is only one example of VHH production in filamentous fungi [11]. It is important to highlight that for oral therapy a pre-requisite is the elimination of any living organism that could contaminate or replicate in the intestinal tract of patients, as well as the removal of any potential contaminating toxin that may aggravate the diarrhea. Thus the production of these antibodies in bacteria could introduce some of the mentioned undesirable factors into the final product, even after several purification steps. Discarding bacteria fermentation for antibody production removes the option of one of the most cost-efficient production systems available. Therefore the only possibility to apply the above mentioned VP6-specific VHHs as a preventive or therapeutic strategy to control RV A in developing countries is to minimize their production costs. To achieve this goal, it is critical to explore alternative expression platforms that present less risk in terms of undesirable contaminants of the final products, considering the disease and administration route used. It is noteworthy that N-glycosylation of some VHHs contributes to toxin- and virus-neutralizing capacity [12]. The use of eukaryotic expression systems would therefore be advantageous for VHH production. The therapeutic concept of protective single-domain antibodies against RV A could be envisaged not only as a conventional medicament but also as an additive to elaborate a functional food able to reduce the incidence or severity of severe RV A diarrhea, especially in risk populations (infants in day care centers, hospitalized children, immunosuppressed patients, etc.). Therefore, the choice of an efficient heterologous expression system, involving high production efficiency or downstream processing, would represent a step towards the clinical application of VHHs.

The baculovirus expression vector system (BEVS) is a widely used method to efficiently produce functional recombinant proteins, including any kind of antibodies. However, the production cost of BEVS is relatively high when insect cells lines are used because the cell culture characteristics and linear scalability is complex for therapeutic proteins. In addition, the investment in bioreactors is potentially too high when the molecule to be developed is directed mainly at the markets of developing countries and will probably be used at high therapeutic doses administered by the oral route. An economically efficient alternative to insect cell cultures is the use of insects as living biofactories. Numerous studies have demonstrated the cost-efficiency and the scalability of production of many recombinant proteins, which include diagnostic reagents [13-17], vaccines [14,18-26] and therapeutic molecules [27], among others. The most used insect larvae for such purposes is the natural host of *Autographa californica multiple nucleopolyhedrovirus* (AcMNPV), the lepidopter *Trichoplusia ni* (*T. ni*). Recombinant protein production by the combination of recombinant baculovirus and this insect larva has been denominated improved baculovirus expression system (IBES®). This technology represents one of the best production alternatives based on baculovirus vectors. Baculovirus-infected larvae produce high yields of recombinant protein (frequently more than one milligram per insect) at a reduced cost [28] and working in non-sterile conditions. These larvae provide a final product with high biosafety standards as animal compounds are not involved in the production process and neither does cross-infection in mammals occur between pathogens infecting this lepidopter [18,20,21,26,29].

Here we successfully applied IBES® technology to express two VHH antibodies, 2KD1 and 3B2, that show neutralizing and protective activity against various strains of RV A [5]. Using low recombinant baculovirus doses to express the VHHs, we produced 1.4 and 1.8 mg/ g of insect biomass of recombinant 3B2 and 2KD1, respectively (up to 257 mg/L of insect extract). The larva-derived antibodies were fully functional and they neutralized the virus *in vitro* and *in vivo*, in a similar way as the *E. coli*-derived molecules did*.* To the best of our knowledge, this is the first time that insects have been used as living biofactories to produce a VHH molecule.

## Results

### Analysis of VHH expression in T. larvae

**Figure 1:**
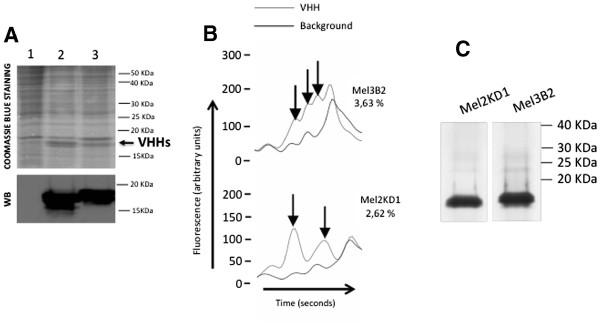
**Expression of recombinant VHHs in*T.ni*larvae.A.** SDS-PAGE analysis and Coomassie blue staining or Western blot (WB) of total soluble protein extracts of larvae infected with control baculovirus (lane 1), baculovirus expressing VHH Mel2KD1 (lane 2) or baculovirus expressing VHH Mel3B2 (lane 3). Western blot was carried out using an anti-VHH polyclonal antibody. The arrow indicates VHH bands. **B.** Analysis by capillary electrophoresis (Experion; Bio-Rad) of larvae extracts containing VHHs 3B2 and 2KD1. The arrows indicate the peaks used to determine the percentage of each recombinant VHH by subtracting the background proteins from the larvae extracts. **C.** SDS-PAGE analysis and Coomassie blue staining of recombinant VHHs purified by affinity chromatography.

The capacity of 3B2 and 2KD1 VHH antibodies to recognize and neutralize RVs belonging to distinct subgroups and serotypes (different G/P-type combinations) *in vitro* and *in vivo* has been addressed previously [5]. To study the feasibility of expressing these potential therapeutic molecules against RV A using the IBES® technology, we generated and characterized two recombinant baculoviruses expressing these antibodies (BacMel3B2His and BacMel2KD1His). The encoding genes for the antibodies were cloned in phase with the mellitin signal peptide to increase productivity, following previous descriptions in the literature. We inoculated groups of 100 fifth instar larvae with a range of doses of each recombinant baculovirus inoculum. The optimal dose selected for VHH production was 50,000 plaque forming units per larvae (pfu/larva) for 3B2 while for 2KD1 it was 10,000 pfu/larva. The larva-derived recombinant antibodies were detected in TSP fractions obtained from infected larvae by Coomassie blue staining of SDS-PAGE gels and showed the expected electrophoretic mobility (Figure 1A). Both VHHs expressed in larvae were also recognized specifically by an anti-VHH polyclonal antibody in a Western blot (Figure 1A).

Capillary electrophoresis using the Experion system (Bio-Rad) was used to quantify in triplicate the recombinant VHHs produced in optimal conditions in the TSP fractions obtained from larva infected with each recombinant baculovirus. The percentages of recombinant VHHs produced were 2.62 and 3.63 for 2KD1 and 3B2 respectively (Figure 1B). Recombinant VHHs were purified by affinity chromatography (Figure 1C) and recovery rates of around 50% were achieved for both molecules.

#### *In vitro* functionality of the larva-derived VHHs

**Figure 2:**
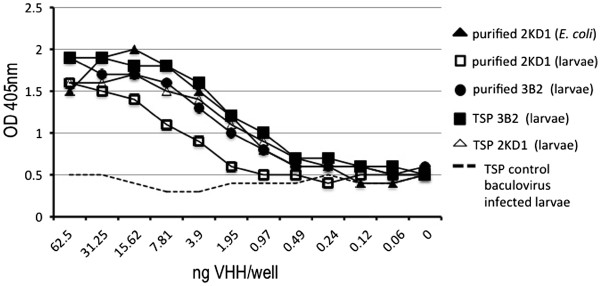
**ELISA detection of rotavirus strain INDIANA (SbI, P**[5]**G6) using larva-derived VHHs.** Serial dilutions of purified or raw material from larvae expressing monomeric VHH 3B2 or 2KD1 were tested and compared with the VHH obtained from *E. coli*. The cut-off point of the assay was established at an OD405nm of 0.0664.

We first analyzed the recognition of the VP6 protein in the context of the RV A particles obtained in infected cell cultures. Virus supernatant containing 10^7^ FFU/ml from MA-104 cells infected with the bovine RV INDIANA (SbI, P[5]G6) was used as antigen in an ELISA. Larva-derived VHHs (purified 3B2 or 2KD1, 3B2 TSP and 2KD1 TSP) recognized the viral particles in a similar way to the purified 2KD1 VHH expressed in *E. coli,* which was used as a positive control (Figure 2).

**Figure 3:**
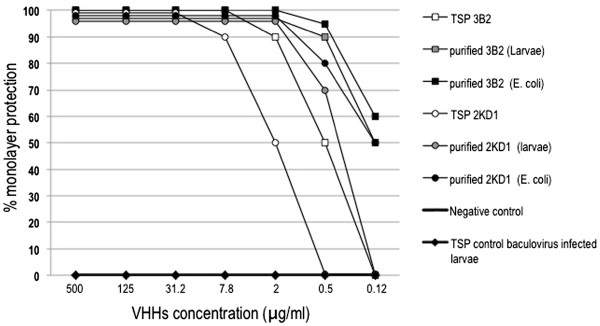
***In vitro*RV neutralization properties of larva-derived single-domain antibodies determined by a fluorescent focus reduction assay.** Different amounts of VHHs were mixed with 100 fluorescent focus-forming units (FFU) of human RV strain Wa (SbI, P [8]G1). The antibody concentration that generates an 80% reduction of fluorescent focuses was considered protective. The assay also included 3B2 and 2KD1 purified from *E. coli* as positive controls and the total soluble protein of unrelated baculovirus-infected larvae as a negative control.

In order to evaluate the neutralization activity of larva-derived VHHs, recombinant single-domain antibodies were analyzed in a VN test using serial four-fold dilutions of antibodies and the Wa human RV strain (SbI, P[8]G1). Similar patterns of cell protection were obtained along the serial dilutions tested for all the VHHs analyzed. The two VHHs purified from larvae, 3B2 and 2KD1, neutralized more than 90% of RV infection *in vitro* at similar concentrations to their *E. coli* counterparts ( 0.5 and 2 μg/ml, respectively) (Figure 3).

When raw extracts from larvae containing 3B2 or 2KD1 were assayed in VN assays, low concentrations of 2 μg/ml or 7.8 μg/ml of antibody, respectively, were also required to neutralize 90% of virus infectivity. This observation suggests that other components of the insect included in the samples assayed did not interfere with the functionality of the VHHs (Figure 3).

### *In vivo* functionality of larva-derived VHHs

**Figure 4:**
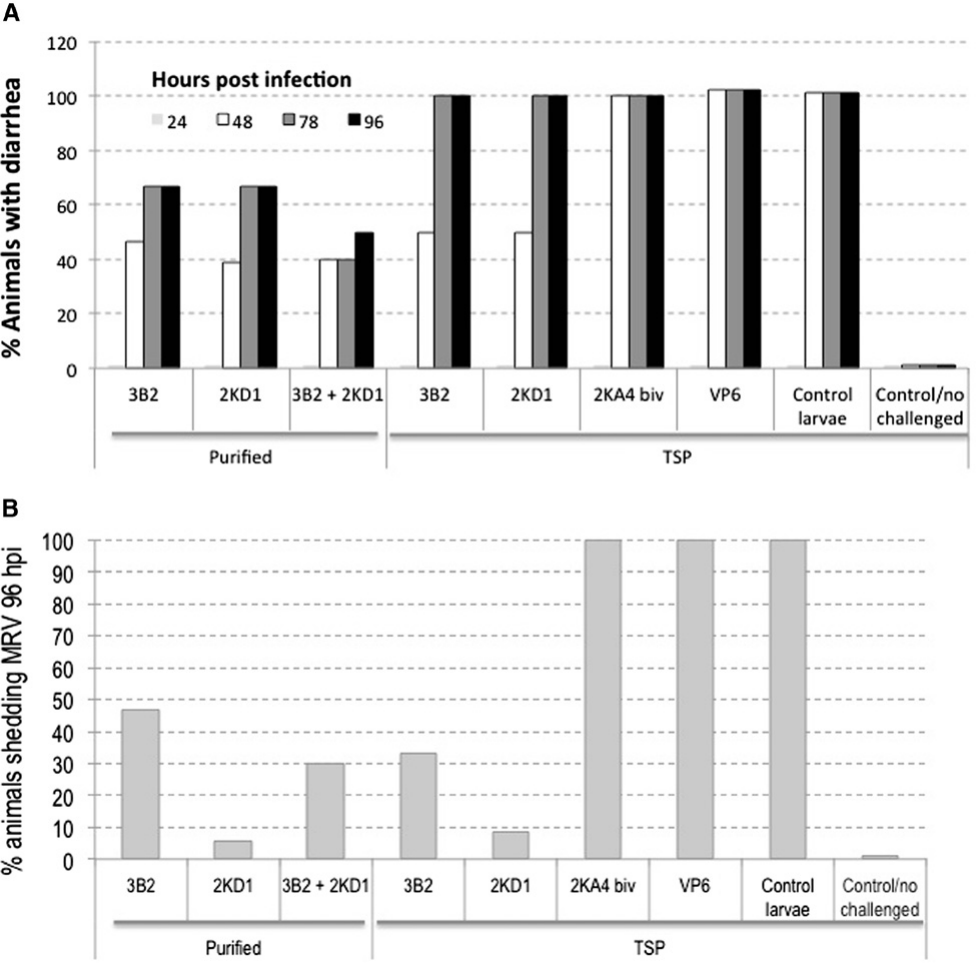
**Protection against diarrhea and prevention of viral shedding achieved by oral administration of monovalent larva-derived VHHs 2KD1 or 3B2 in suckling mice challenged with RV.** Pups were fed 100 μg (100 μl) of each larva-derived VHH intragastrically from days 0 to 5 once a day. At day 1, the pups were challenged intragastrically with RV 2 h after routine feeding. **A.** Percentage of pups protected from diarrhea with 300 DD50 of murine RV ECw at a range of post-infection days. **B.** Viral shedding was quantified by ELISA in the 10% (wt/vol) small intestine homogenates. The Fisher exact test was used to compare the proportions of pups with diarrhea and viral secretion among the groups. Antibody 2KA4 (divalent VHH antibody) or VP6 protein, both also produced in larvae, were used as controls.

For a further demonstration of the functionality of VHHs produced in larva, we performed *in vivo* protection experiments against RV A in newborn mice. Pups received a single intragastric dose of 100 μg of each purified VHH (3B2 or 2KD1) daily for 5 days. On the second day of VHH administration, animals were challenged with the murine RV strain ECw, which produced diarrhea in 100% of the untreated control mice. Challenged pups developed RV-associated diarrhea 48 h post challenge (hpc). Non-challenged animals fed TSP from control infected larvae developed no clinical sign during the experiment, thus indicating that the components of the larvae extracts did not induce diarrhea per se. At the time point corresponding to the onset of diarrhea, the rate of diarrhea among groups was statistically analyzed. All groups of mice treated with neutralizing VHHs (purified or TSP) showed significantly higher protection rates against RV-associated diarrhea than the control groups (treated with 2KA4 non-neutralizing VHH, larvae extracts containing the irrelevant recombinant protein VP6 or TSP from control baculovirus-infected larvae), which presented severe diarrhea at 48 hpc in 100% of pups (Figure 4). Specifically, the pups treated with purified 3B2 and 2KD1 VHHs showed significantly higher protection rates against diarrhea (53% and 61%, respectively) than the control groups (0%) at 48 hpc. In addition, the protective effect was maintained until the end of the experiment (96 hpc), where 33% of the treated mice in each group remained without diarrhea (Figure 4A).

To evaluate the protective effect of the VHH treatment against viral shedding, at the end of the *in vivo* protection assays (96 hpc) all pups were euthanized and intestine homogenates were checked by ELISA. Results were statistically analyzed. Similarly to what was observed for clinical signs, all groups of pups fed neutralizing VHHs showed significantly lower rates of viral shedding than the control groups (Figure 4B). In agreement with the diarrhea protection, only 53% of pups fed purified 3B2 VHH did not show viral shedding at 96 hpc, while pups fed the purified 2KD1 VHH showed a significant reduction rate of viral shedding compared with the control groups as well as with 3B2, where only 6% (1 out of 18) were positive for RV A by ELISA (Figure 4B).

In a second experiment, we examined whether there was a synergistic protective effect of both larva-derived purified 3B2 and 2KD1. Interestingly, 60% (4 out of 10) of the pups treated with 50 μg/dose of each VHH (3B2 + 2KD1) were fully protected against diarrhea at 48 and 78 hpc and 50% (5 out of 10) remained without any clinical sign at 96 hpc (Figure 4A). Regarding viral shedding, only 30% of the animals (3 out of 10 mice) presented virus in intestine samples, showing an intermediate rate of viral shedding between pups fed each VHH alone (47% and 6%, respectively) but still representing a significant reduction compared with the control groups (100%).(Figure 4B),

With the aim to determine whether raw material from larvae containing the 3B2 or 2KD1 antibodies was also protective in mice, two groups of pups were treated with these extracts, which held identical amounts of VHHs as tested in the previous experiment using purified antibodies. In this experiment, the number of pups protected at 48 hpc was lower (50% for neutralizing 3B2 and 2KD1). All animals presented clinical signs of diarrhea at later times post infection (Figure 4). In addition, both groups showed a statistically significant reduction of viral shedding at 96 hpc, with protection rates of 66.7% (8 out of 12 mice) for 3B2 and 91.7% (11 out of 12 mice) for 2KD1 (Figure 4B).

## Discussion

Many novel therapeutic molecules are based on antibodies directed to molecular targets, including infectious pathogens. These therapeutic antibodies can be produced in a number of ways while maintaining their antigen affinity properties. However, conventional antibodies are frequently difficult to produce because of their complex structure. Short versions, such as single-chain antibodies, comprising the antibody-binding domains, lose some antibody properties and present reduced affinity and stability. Single-domain antibodies produced by *camelidae* have very interesting properties compared to classical mammalian antibodies as they have only one variable region (heavy chain), with a reduced size, and show greater stability under a range of conditions (Conrath et al., 2005). By a mechanism that is not fully elucidated and probably related to the small size of 3B2 and 2KD1, the interaction of these antibodies with their target antigen (the highly antigenically preserved viral protein VP6) in RV particles differs to that of conventional immunoglobulins. Thus, 3B2 and 2KD1 produce broad neutralization activity *in vitro* and are protective against diarrhea and viral shedding in a neonatal mouse model. These properties confer these antibodies a high potential therapeutic value. Experiments to demonstrate the molecular basis of this mechanism of interaction are currently under way.

Monoclonal antibodies are crucial in biopharmaceutical development and account for 42% of the total market of biologics. The annual growth of market requirements is estimated to increase to as much as 25 tons of therapeutic antibodies per year in 2013. This value would correspond to an annual growth rate of 37% [30]. This scenario has driven pharmaceutical companies to identify novel production alternatives to conventional fermentation technologies that facilitate speed of development and production scale-up at reduced costs. In the last 15 years, living biofactories such as plants, transgenic mammalians and insects have been adapted to produce recombinant proteins of all kinds, providing excellent results. Today, some of these methodologies represent true alternatives to the fermentation of bacteria, yeast, and mammalian or insect cells.

Insect larvae frequently scale up the recombinant protein production mediated by baculovirus vectors to levels of grams/L or hundreds of grams/L instead of tens of milligrams/L usually obtained in insect cells under fermentation. This technology dramatically reduces the production costs and fixed investments while also offering other advantages over classical insect cell fermentation approaches. First, insects are complex organisms that contain multiple cell types in perfect physiological conditions for productivity. Complexity frequently increases productivity in any expression system. Second, the scale-up of production based on insects (lepidopters) is unlimited and fast. Tons of silk thread, composed by two proteins, are produced by a lepidopter every year and companies using insects to produce baculoviruses as biopesticides generate millions of infected insects in short periods of time by simple procedures. Third, given the flexibility of baculovirus vectors, the development times implied by use of insects as living biofactories is very short when compared to the generation of productive transgenic mammalian cell clones.

The first descriptions of insect larvae use as living biofactories emerged in the early ‘90s [31] and several companies in Europe, USA and Asia currently exploit this technology using *Trichoplusia ni* or *Bombyx mori* lepidopters with their respective baculovirus vectors. Various kinds of protein have been successfully expressed in larvae, including diagnostic reagents [13-17], vaccines [14,18-26,29], therapeutic proteins [27], enzymes [32,33], and conventional complete or fragmented antibodies [34,35]. However, to the best of our knowledge, this is the first description that evaluates the efficiency of insects as living biofactories to express recombinant single-domain antibodies (VHH) derived from llama. Our results demonstrate that insects are an efficient alternative to produce fully functional VHHs, which, in this case, can be used for diagnostic and therapeutic purposes. The antibodies 3B2 and 2KD1 are directed to unknown epitopes within the VP6 inner capsid protein, which is highly immunogenic and conserved among all Group A RVs, including several subgroup specificities, various G/P-type combinations, and distinct species of origin, as previously demonstrated [5].

These VHH molecules were previously generated functionally in *E. coli*. However, the use of insect host cells to produce VHHs would provide further advantages, such as the possibility to make posttranslational modifications, including the formation of disulphide bonds [36]. In this case, llama VHHs, which have an extended CDR3 that is often stabilized by an additional disulphide bond with a cysteine residue in CDR2 [1,37], would be more efficiently produced.

The VHHs expressed in this study were cloned transcriptionally in phase with the signal peptide of honey bee mellitin. The incorporation of this signal peptide to the construct increased the expression yields, as previously reported with other proteins in the baculovirus expression system [38], in comparison to the use the KDEL ER retention sequence (at least 200% increase). This signal peptide would allow the glycosylation of the protein. Although an anomalous migration in SDS-PAGE was observed in the VHH 3B2, this characteristic does not seem to be caused by this modification more than by another post-transductional modification since no glycosylation was detected when using the GLYCOPRO Detection Kit (Sigma, USA) (data not shown).

The binding capacity of the larva-derived 3B2 and 2KD1 VHHs was compared in a sandwich ELISA with the same antibodies expressed in *E. coli*. The binding behavior was similar in all cases with respect to the RV viral particles (Figure 2). In addition, both molecules neutralized Group A RV strain Wa (SbI, P [8]G1) when they were incubated with the virus (Figure 3). The capacity of the monomeric VHH to present neutralizing activity could be related to the small size of these molecules [5]; however, research into the mechanism behind the RV virus neutralization is still underway.

Furthermore, the intragastric administration of the VHH 3B2 or 2KD1 in a neonatal mouse model exerted a protective effect against RV A-induced diarrhea caused by a highly infectious murine RV, ECw. Fifty-three percent of the pups treated with purified 3B2 were protected after 48 hpc and at 96 hpc about 35% of the animals were still protected, and none of these protected animals presented viral excretion. With the purified 2KD1, 61% of the animals were protected against diarrhea at 48 hpc and only one pup showed viral shedding 96 hpc, resulting in the group with the highest protection rates against RV infection and disease. Using non-purified larvae crude extracts containing the VHHs object of this study (TSP), protection levels were lower compared with their purified counterparts. However, 66.7% and 91.7% of the animals presented no viral excretion at 96 hpc by ELISA when treated with 3B2 TSP or 2KD1 TSP, respectively (Figure 4). These results were very similar to those previously obtained when using the same VHHs expressed in *E. coli*[5]. The most interesting protective results were obtained when the 3B2 and 2KD1 VHHs were combined, since 60% of the animals remained protected at 78 hpc and 50% after 96 hpc. Viral shedding was also dramatically reduced to 30% in this group (Figure 4). Our results encourage us to continue to evaluate the protective value of these VHHs in a larger animal model of RV infection and disease that more closely resembled the physiology of human infants. Experiments with the larva-derived VHHs described here in gnotobiotic pigs are in course.

In conclusion, the monovalent VHHs were efficiently expressed in larvae and presented the same functionality as those expressed in *E. coli*, showing polyreactive properties against several RV strains (INDIANA (SbI, P[5]G6), Wa (SbI, P[8]G1) and Ecw) both in ELISA and VN. These properties were shown to be associated with protection in a neonatal mouse model. Our results open up the possibility of using insects as living biofactories for the cost-efficient production of these and other VHHs to be used for diagnostic or therapeutic purposes, thereby eliminating concerns regarding the use of bacterial or mammalian cells.

## Conclusions

Control or therapeutic molecules against severe infant diarrhea produced by rotavirus A are of tremendous interest due to the number of infection episodes per year (more than 111 million cases), mainly in children in developing countries. Two VHHs, named 3B2 and 2KD1, specific for the inner capsid protein VP6 of Group A rotavirus have been previously characterized as protective in a rotavirus infection mouse model. A cost-efficient production is a prerequisite to apply these single-domain antibodies as a preventive or therapeutic strategy to control rotavirus A infections in developing countries. In this work we have modelized the expression of the above mentioned VHHs by the Improved Baculovirus Expression System (*IBES*® technology). This technology uses a baculovirus expression vector in combination with *Trichoplusia ni* larvae as living biofactories. The reached expression levels of VHHs represented up to 257 mg/L of protein extract after insect processing. The larva-derived antibodies were fully functional when tested *in vitro* and *in vivo*, neutralizing Group A rotaviruses and protecting offspring mice against rotavirus-induced diarrhea. The *IBES*® technology has the potential to eliminate many concerns regarding the use of bacterial or mammalian cells, and to our knowledge, this is the first time that insects have been used as living biofactories to produce a VHH molecule.

## Methods

### Construction of recombinant baculoviruses expressing VHH

The donor vectors were generated as follows, 3B2 or, 2KD1 were PCR-amplified from previously described and tested pHEN plasmid containing VHH clones of interest (Garaicoechea et al., 2008) and using the following as specific primers:

pVHH1: 5^′^…GCTTGGATCCTATGGCTGATGTGCAGCTGC…3^′^

pVHH2: 5^′^…CGTATCTAGAGCGGCCGCGTGAGGAGACG…3^′^

pVHH3: 5^′^…ACTGTCTAGAGCGGCCGCTGGAGAC…3^′^

pVHH1 and pVHH2 were used for amplification of the 3B2 VHH, and pVHH1 and pVHH3 for the 2KD1 VHH.

The amplicons were then cloned into the pFastMelB2 vector in frame with an insect signal sequence derived from the honey bee, using the *BamH*I and *Xba*I restriction sites included in the corresponding primers.

The recombinant baculoviruses BacMel3B2His and BacMel2KD1His, encoding the VHH molecules 3B2 and 2KD1 respectively, were obtained using the Bac-to-Bac™ baculovirus expression system (Invitrogen), following the manufacturer’s instructions. All constructs incorporated a His tag sequence at the carboxy terminus of the protein to facilitate antibody purification.

#### Infection of insect larvae and Sf21 insect cells

*Trichoplusia ni* larvae were reared under level 2 biosafety conditions, as previously described [17]. Groups of 100 fifth instar larvae, between 140–170 mg in weight, were intraemocelically injected with 5 μl of the doses of the different recombinant baculovirus inocula. Larvae injection was performed with disposable syringes and using a hand microapplicator (Burkard Co.UK). Inoculated larvae were kept at 28°C in specifically designed methacrylate modules for insect rearing and collected after 72 h post-infection (hpi). A total of 20 repetitions per treatment (2,000 larvae) were made. The surviving infected larvae collected per container were counted and weighed together to obtain the total biomass recovered per infection dose. Larvae were then frozen immediately at −28°C until processing. *Spodoptera frugiperda* (Sf21) were obtained from Invitrogen. This insect cell line derives from the ovarian tissue of the fall armyworm *Spodoptera frugiperda*. Sf21 were routinely grown in TNMFH medium (PAN Biotech) supplemented with Foetal Bovine Serum 10% (PAN Biotech, 3302-P101304) and gentamycin sulfate 50 mg/ml (Lonza, BE02-012E).

#### Insect protein extract preparation, characterization and recombinant protein purification

Total soluble protein (TSP) from infected larvae (30 μg/lane) was extracted as previously described (Perez-Filgueira et al., 2006) and was analyzed by 15% SDS-PAGE and stained with Coomassie blue or transferred onto nitrocellulose membranes to perform a Western blot using a polyclonal anti-VHH antibody generated in rabbits [5] at 1:200 dilution in blocking solution (4% fat free dry milk in PBS 1X) for 1 h, followed by one wash with PBS 1X for 15 min and two more washes for 5 min. A secondary incubation was then carried out for 1 h with an anti-rabbit IgG, horse radish peroxidase (GE Healthcare) at 1:2000 dilution in blocking solution. Membranes were developed using ECL reagent and images were capture by Chemidoc (Bio-Rad) and analyzed using the Image lab^TM^ Software, version 2.0.1 (Bio-Rad).

For quantification, the specific samples were loaded in Pro260 chips (Bio-Rad) and analyzed by capillary electrophoresis using the Experion system (Bio-Rad), following the manufacturer’s instructions. The Experion system resolved and quantified protein samples from 10 to 260 kDa in size with high sensitivity comparable to colloidal Coomassie blue SDS-PAGE gel staining. Each amount (percentage) of recombinant protein was obtained by means of direct calculation of the area corresponding to the protein in the specific TSP extract profile. The control protein extract (Ni) was used as a “background” subtracted from the estimated area.

### Enzyme-Linked ImmunoSorbent Assays (ELISAs) for VHH characterization

#### Group A RV ELISA

Sandwich ELISA. Maxisorp plates were coated with a 1/10,000 dilution of a polyclonal antiserum to RV A obtained from a gnotobiotic RV-infected pig (kindly supplied by Dr. LJ Saif, OSU, USA). After blocking plates with 4% semi-skimmed milk in PBS at pH 7.4, we added 10^7^ FFU/ml of the bovine RV A reference strain INDIANA (SbI, P[5]G6) or mock-infected MA-104 cells (used as negative control) to the wells. Plates were blocked with 10% skimmed milk in PBS at pH: 7.4. Serial two-fold dilutions of the recombinant VHHs were added and incubated at 37°C for 1 h. Plates were washed four times in PBS at pH 7.4, 0.05% Tween20_,_ and the VHHs were detected by incubation for 1 h with a rabbit polyclonal anti-VHH (1:7000 diluted in PBS pH 7.4/ 0.05% Tween20/ 2% skimmed milk solution). ELISA was then revealed using a horseradish peroxidase-conjugated IgG anti-rabbit (KPL) and H_2_O_2_/ABTS as substrate/chromogen system. The background (signal given by the mock-infected cells) was subtracted from each sample. The cut-off point for the assay was established as mean plus three standard deviations of the corrected optical density (OD) measured in the PBS wells (blank). Using this cut-off, the detection limit of the assay was 0.5 to 0.2 ng/well of functional VHH specific for RV A.

#### Viral neutralization assay (VN test)

The fluorescent focus neutralization test was used for purified VHH molecules, as described previously [39]. Briefly, serial four-fold dilutions of purified VHH molecules were mixed with 100 fluorescent focus-forming units (FFU) of human RV strain Wa (SbI, P[8]G1), followed by incubation for 1 h at 37°C. Mixtures were then plated onto MA-104 monolayers and incubated for 48 h at 37°C. The plates were fixed with 70% acetone, and RV replication was detected using a fluorescein-labeled anti-RV antiserum made in bovine at a 1/100 dilution. The neutralizing activity of VHH is expressed as the minimum amount of recombinant protein required to decrease the fluorescent foci produced by 100 FFUs by 80%.

#### Rotavirus protection assay in neonatal mice

Assays were carried out as previously described [5]. Basically, groups of four-day-old BALB/c mouse pups received 100 μl of a solution containing 100 μg of recombinant VHH per dose, via an intragastric gauge, once a day for 5 days, starting on day 0. On day two of the VHH treatment, pups received 20 μl of 5% bicarbonate solution followed by intragastric challenge with 300 DD50 of murine RV strain ECw (a dose causing diarrhea in 100% of control mice). RV-inoculated mice not treated or treated with VP6 larva extract, and mice treated with the same amount of a non-neutralizing bivalent 2KA4 VHH were included as controls. Mice fed larvae infected with wild-type baculovirus and challenged with murine RV or not challenged were also included in order to discard the possibility that the larvae extracts themselves induced diarrhea. Challenges were conducted in two independent experiments. To detect induction of diarrhea, pups were clinically evaluated by direct palpation of the abdomen by a well-trained person working in a blinded fashion with regard to the treatment groups. As murine RV ECw spread in littermates, cases of diarrhea that developed 24 h later or more after the first case in a litter were not attributable to the initial inoculum and therefore were not considered. At 4 days (96 h post challenge), pups were euthanized and small intestines were removed, frozen-thawed, and homogenized in 10% (wt/vol) Hanks balanced salt solution (HBSS). The homogenates were assayed for the detection of RV by ELISA (Cornaglia et al., 1989). The Fisher exact test was used to compare the proportions of pups with diarrhea and viral shedding between the experimental groups. Statistical significance was assessed at p < 0.05.

All animal experiments were performed in accordance with protocols approved by the Instituto de Virología (CICVyA) and the INTA Ethical committee (CICUAE) as stated in the resolution N13/2011.

## Competing interests

SGS, CNS, CA and RL are employees of Alternative Gene Expression S.L. (ALGENEX) company. JME is co-founder and stockholder and is a paid consultant to ALGENEX. ALGENEX is proprietary of the IBES® technology and has the world exclusive license for the application of the patented VHHs object of this work for therapeutic and diagnostic purposes. LG, AW, JME, SGS and VP are authors of the patent covering the application of the VHHs 3B2 and 2KD1 as therapeutic and diagnostic molecules. MM and AK report no conflicts of interest.

## Authors’ contributions

SGS and CMN performed the cloning and generation of recombinant baculoviruses for VHHs expression, characterized the recombinant antibodies and their function *in vitro.* SGS drafted the manuscript. CA purified and quantified the larva-derived antibodies. RL coordinate the recombinant VHHs production by *IBES*® technology. MM, LG and AK characterized the neutralization and *in vivo* protection activities of larva-derived VHHs. AW and VP conceived and coordinated the studies of functional activities of VHH antibodies. JME conceived the study, participated in its coordination and projection, and edited the manuscript. All authors have read and approved the final manuscript.
